# cfTools: an R/Bioconductor package for deconvolving cell-free DNA via methylation analysis

**DOI:** 10.1093/bioadv/vbaf108

**Published:** 2025-05-06

**Authors:** Ran Hu, Shuo Li, Mary L Stackpole, Qingjiao Li, Xianghong Jasmine Zhou, Wenyuan Li

**Affiliations:** Department of Pathology and Laboratory Medicine, David Geffen School of Medicine, University of California at Los Angeles, Los Angeles, CA 90095, United States; Bioinformatics Interdepartmental Graduate Program, University of California at Los Angeles, Los Angeles, CA 90095, United States; Institute for Quantitative & Computational Biosciences, University of California at Los Angeles, Los Angeles, CA 90095, United States; Department of Pathology and Laboratory Medicine, David Geffen School of Medicine, University of California at Los Angeles, Los Angeles, CA 90095, United States; Department of Pathology and Laboratory Medicine, David Geffen School of Medicine, University of California at Los Angeles, Los Angeles, CA 90095, United States; Department of Pathology and Laboratory Medicine, David Geffen School of Medicine, University of California at Los Angeles, Los Angeles, CA 90095, United States; Department of Pathology and Laboratory Medicine, David Geffen School of Medicine, University of California at Los Angeles, Los Angeles, CA 90095, United States; Institute for Quantitative & Computational Biosciences, University of California at Los Angeles, Los Angeles, CA 90095, United States; Department of Pathology and Laboratory Medicine, David Geffen School of Medicine, University of California at Los Angeles, Los Angeles, CA 90095, United States

## Abstract

**Motivation:**

Cell-free DNA (cfDNA) released by dying cells from damaged or diseased tissues can lead to elevated tissue-specific DNA, which is traceable and quantifiable through unique DNA methylation patterns. Therefore, tracing cfDNA origins by analyzing its methylation profiles holds great potential for detecting and monitoring a range of diseases, including cancers. However, deconvolving tissue-specific cfDNA remains challenging for broader applications and research due to the scarcity of specialized, user-friendly bioinformatics tools.

**Results:**

To address this, we developed cfTools, an R package that streamlines cfDNA tissue-of-origin analysis for disease detection and monitoring. Integrating advanced cfDNA tissue deconvolution algorithms with R/Bioconductor compatibility, cfTools offers data preparation and analysis functions with flexible parameters for user-friendliness. By identifying abnormal cfDNA compositions, cfTools can infer the presence of underlying pathological conditions, including but not limited to cancer. It simplifies bioinformatics tasks and enables users without advanced expertise to easily derive biologically interpretable insights from standard preprocessed sequencing data, thus increasing its accessibility and broadening its application in cfDNA-based disease studies.

**Availability and implementation:**

cfTools and its supplementary package cfToolsData are freely available at Bioconductor: https://bioconductor.org/packages/release/bioc/html/cfTools.html and https://bioconductor.org/packages/release/data/experiment/html/cfToolsData.html. The development version of cfTools is maintained on GitHub: https://github.com/jasminezhoulab/cfTools.

## 1 Introduction

DNA methylation is a stable epigenetic modification that is unique to differentiated cells (Moore *et al.* 2013). In pathological conditions such as cancer, tumor cells often exhibit abnormal DNA methylation patterns that deviate from their tissue of origin but consistent with the progression of tumor (Manel 2008). Circulating cell-free DNA (cfDNA) in blood are short DNA fragments released from various tissues, including tumors in cancer patients (Wan *et al.* 2017, Huang and Wang 2019). This suggests that cfDNA molecules possess methylation patterns that not only indicate their tissue of origin but may also exhibit aberrations originating from tumors. Studies have demonstrated that deciphering the tissue composition of cfDNA through methylation profile has promising clinical application in noninvasive diagnosis and prognosis (Sun *et al.* 2015, Barefoot *et al.* 2021, Del Vecchio *et al.* 2021) and monitoring disease progression (Li *et al.* 2023). However, there is a lack of comprehensive and user-friendly software toolkits that provide straightforward cfDNA deconvolution from raw or preprocessed sequencing data, allowing for the sensitive detection and accurate quantification of trace amounts of tissue-specific or tumor-derived cfDNA from various origins in a single workflow.

Here, we present cfTools, an R/Bioconductor package that provides a seamless workflow from standard Bismark-processed files to downstream deconvolution functions that decipher cfDNA tissue-of-origin based on methylation profiles (Li *et al.* 2018, Del Vecchio *et al.* 2021, Li *et al.* 2023). Through the analysis of methylation patterns of tumor or tissue reference markers at the individual DNA fragment level, cfTools calculates the proportions of cfDNA fragments derived from tumor or normal tissues, thereby informing the global tissue composition of plasma cfDNA. Building on our previously proposed methods developed in Python and C++, cfTools integrates them into R, providing easy access to all the methods within a single tool and ensuring full compatibility with the Bioconductor environment. The package features data preparation functions and data analysis functions with flexible parameter options to enhance user-friendliness and accommodate the diverse needs of users. To distinguish tumor-derived cfDNA from background fragments and estimate tumor burden, it provides the *CancerDetector* (Li *et al.* 2018) function. To deconvolve multiple tissue types and quantify the tissue composition of cfDNA, it provides two functions. One is *cfDeconvolve*, which is an unsupervised probabilistic approach (Del Vecchio *et al.* 2021) that can incorporate any user-defined methylation markers and various types of methylation data. The other function is *cfSort* (Li *et al.* 2023), a supervised deep learning-based pretrained model designed to work with predetermined methylation markers of 29 tissue types and cfDNA methylome sequencing data, such as Whole-Genome Bisulfite Sequencing (WGBS) and cfDNA Methylome Sequencing (cfMethyl-Seq) (Stackpole *et al.* 2022) data. While the supervised method offers enhanced accuracy with predefined markers, the unsupervised method provides greater flexibility by accommodating user-specified markers and an unknown tissue type but may not achieve the same level of precision. In addition, to facilitate the use of cfTools, we created the cfToolsData package that offers users access to reference marker files, which encompasses DNA methylation marker information for four cancer types (colon, liver, lung, and stomach cancer) (Stackpole *et al.* 2022) and 29 primary human tissue types (Li *et al.* 2023). Featuring user-customizable options, cfTools and cfToolsData facilitate the straightforward extraction of biologically meaningful information from standard preprocessed sequencing data, accommodating a wide range of clinical applications such as disease diagnosis, prognosis, and monitoring.

## 2 Description of cfTools

**Figure 1.**
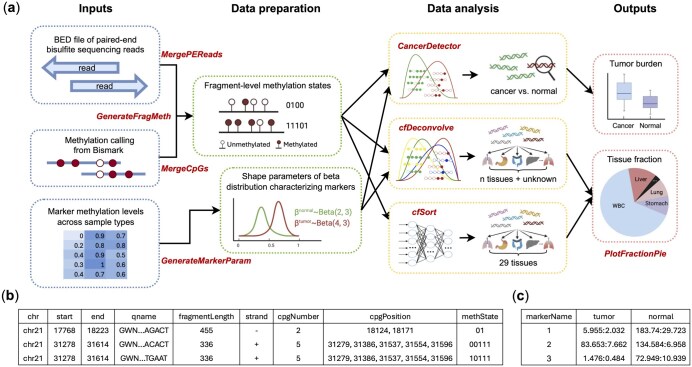
The cfTools workflow and key data files. (a) The main workflow of cfTools encompassing data processing and downstream analysis. The data preparation functions for generating fragment-level methylation states take standard output files from Bismark as inputs. To generate marker parameter files, methylation levels of markers across a list of samples are required. *CancerDetector* and *cfDeconvolve* statistically model the methylation patterns of cancer or tissue marker to deconvolve tumor-derived or tissue-specific fragments, thereby estimating tumor burden or tissue fraction of user-defined tissue types. *cfSort* infers the composition of 29 predefined tissues from the fragment-level methylation profile of a cfDNA sample. (b) The output file of the *GenerateFragMeth* function contains both fragment information and the methylation states of all CpGs on the fragment. The fragment-level methylation states are in the last column “methState”. (c) The output file of the *GenerateMarkerParam* function contains the paired shape parameters of beta distributions for each marker across different tissues. The parameters of a Beta(α, β) distribution are formatted as “α:β”.

cfTools provides a comprehensive suite of functions for streamlined data processing and analysis (Fig. 1a). It can be easily downloaded and installed through the Bioconductor platform (https://bioconductor.org/packages/release/bioc/html/cfTools.html), ensuring accessibility to a wide community of users.

### 2.1 Input files

To generate the primary input file for fragment-level methylation states, the workflow begins with a standard BED file of paired-end bisulfite sequencing reads derived from Bismark-aligned BAM files, along with methylation calling files (CpG_OT* and CpG_OB*) from Bismark (Krueger and Andrews 2011). The other input file required for *CancerDetector* and *cfDeconvolve* is a marker file containing the methylation patterns of different tissues. Users can either use the reference marker files provided in the cfToolsData package or generate their own.

### 2.2 Data preparation

Generating fragment-level methylation states involves three steps: (i) the *MergePEReads* function merges base calls from the overlapping portions of paired-end reads; (ii) the *MergeCpGs* function merges methylation states for CpGs on paired-end reads from the same fragment; and (iii) the *GenerateFragMeth* function uses the outputs from the previous two steps to produce the final output file (Fig. 1b).

To provide users with easy access to marker files, the cfToolsData package includes DNA methylation marker files for tumor tissues from four cancer types (colon, liver, lung, and stomach) and 29 primary types of normal human tissues. Users can intersect their cfDNA fragments with our reference marker files to identify overlapping genomic regions and then filter the reads to retain only those that map to the given marker regions, thereby improving computational efficiency. If users prefer to generate their own marker files instead of using our reference versions, cfTools offers the *GenerateMarkerParam* function. This function processes methylation levels (e.g. beta values) of markers across a list of samples and converts them into paired shape parameters of beta distributions for these markers (Fig. 1c).

### 2.3 Data analysis

The data analysis functions include *CancerDetector*, *cfDeconvolve*, and *cfSort*. Our algorithms trace the origin of each cfDNA fragment based on the fragment-level methylation rate and utilize the tissue-specific fragment counts to infer the cfDNA composition through either probabilistic methods or machine-learning techniques (Li *et al.* 2018, Del Vecchio *et al.* 2021, Li *et al.* 2023). A detailed description of the core functions and their underlying algorithms is provided in Supplementary Text S1.

To sensitively deconvolve cancer signals, *CancerDetector* utilizes a probabilistic model to jointly analyze methylation states of multiple neighboring CpG sites on a single cfDNA fragment (Li *et al.* 2018). It calculates the likelihood of each fragment originating from tumor or normal classes. As the first method to exploit joint methylation status at the cfDNA fragment level, *CancerDetector* effectively captures the pervasive nature of methylation and amplifies the signal for sensitive detection of tumor-derived cfDNA. Its idea of differentiating and purifying tumor signal at the cfDNA fragment level has been widely adopted (Liu *et al.* 2020, Stackpole *et al.* 2022).

We expanded *CancerDetector*, a two-class tissue deconvolution method, into *cfDeconvolve*, which can deconvolve multiple tissue types (*N *>* *2) by calculating tissue-specific likelihoods based on fragment-level joint methylation states, thereby improving the signal-to-noise ratio (Del Vecchio *et al.* 2021). *cfDeconvolve* accounts for discrepancies between real-world scenarios and predefined tissue categories by including the proportion of an unknown tissue type. Fragments with a fold change below a threshold (e.g. 2) between the top two likelihoods are classified as ambiguous and assigned to the unknown tissue type, while unambiguous fragments are used to calculate tissue fractions. As an unsupervised method, it has the flexibility to incorporate user-defined tissue types, methylation markers, and various types of methylation sequencing data that capture the fragment-level methylation information.

While existing cfDNA deconvolution methods predominantly use unsupervised models (Sun *et al.* 2015, Moss *et al.* 2018, Feng *et al.* 2019, Caggiano *et al.* 2021, Del Vecchio *et al.* 2021, Keukeleire *et al.* 2023, Loyfer *et al.* 2023, Unterman *et al.* 2024), we have recently developed *cfSort*, the first supervised tissue deconvolution method based on a dual DNN ensemble model (Li *et al.* 2023), which outperforms unsupervised approaches. *cfSort* predicts tissue composition across 29 predefined tissue types based on the fragment-level methylation states of a cfDNA sample. It is trained on 295 484 diverse *in-silico* cfDNA samples generated by mixing the Reduced Representation Bisulfite Sequencing (RRBS) data of 521 tissue samples with a variety of ground-truth tissue compositions. The feature profile for each sample is constructed from our fragment-level tissue methylation marker atlas, which encompasses 29 major human tissue types (Li *et al.* 2023). *cfSort* leverages ground-truth tissue compositions from substantial *in-silico* cfDNA data and comprehensive base-resolution tissue methylation signatures to achieve superior performance. As the feature profile covers genome-wide marker positions, it is optimized for cfDNA methylome sequencing data like WGBS or cfMethyl-Seq.

### 2.4 Outputs

The final outputs of the pipeline are the tumor burden θ (i.e. tumor-derived cfDNA fraction) for *CancerDetector* and the cfDNA fractions from different tissue types (tissue fractions) for *cfDeconvolve* and *cfSort*. We also provide a visualization function, *PlotFractionPie*, which generates a pie chart from a vector of cfDNA fractions, enabling users to easily visualize tissue composition and produce publication-quality figures.

## 3 Usage examples

We demonstrated the use of cfTools by presenting results from fragment-level cfDNA deconvolution using cfMethyl-Seq (Stackpole *et al.* 2022) data from plasma samples of 98 lung cancer patients, 27 liver cancer patients, 21 patients with cirrhosis, and 100 healthy individuals. For *CancerDetector*, we incorporated 10 000 hyper-methylated markers specific to lung cancer and another 10 000 specific to liver cancer, identified in our prior work (Stackpole *et al.* 2022). Beta distribution shape parameters for cancer markers were generated from RRBS data of tumor and adjacent normal tissue samples. For tissue deconvolution methods (*cfDeconvolve* and *cfSort*), we utilized the tissue marker atlas (Li *et al.* 2023). Beta distribution shape parameters for tissue markers and reference tissue marker methylation profiles were generated from the RRBS data spanning 29 major human tissue types. All marker parameters and annotation information are available in the cfToolsData package.

### 3.1 Lung cancer detection

**Figure 2.**
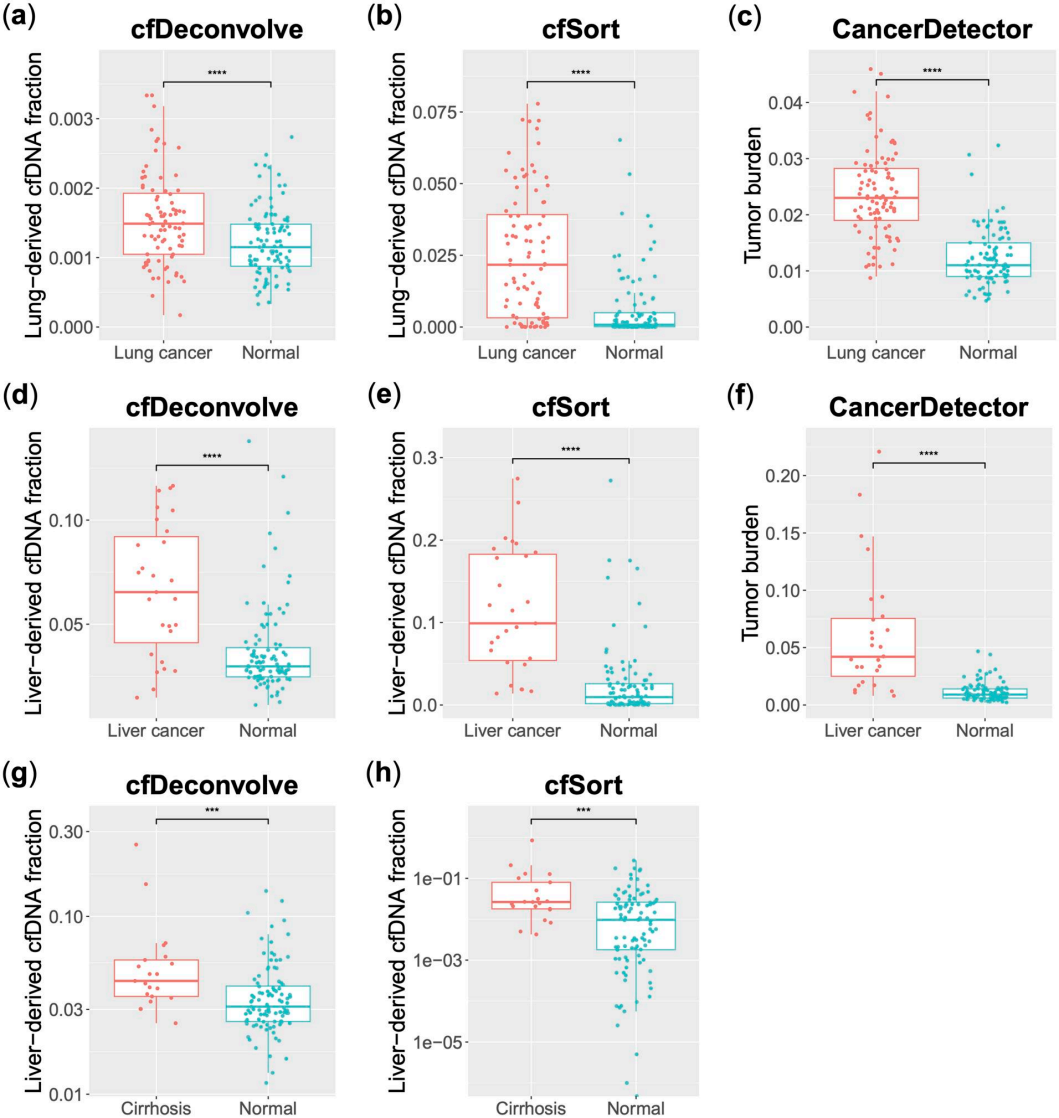
Box plots of diseased tissue-derived cfDNA fraction from *cfDeconvolve* and *cfSort*, and tumor burden from *CancerDetector* in patients and healthy people. (a–c) The estimated lung-derived cfDNA fractions and tumor burden in lung cancer patients versus normal individuals. (d–f) The estimated liver-derived cfDNA fractions and tumor burden in liver cancer patients versus normal individuals. (g and h) The estimated liver-derived cfDNA fractions in patients with cirrhosis versus normal individuals. The distinction between patients and normal individuals was assessed through the Wilcoxon rank-sum tests. The statistical significance of the tests was denoted by the asterisks: “****” means *P* value < .0001, “***” means *P* value < .001.

We used the three core functions to deconvolve plasma samples from lung cancer patients and normal individuals. A substantial increase in both tumor burden and lung-derived cfDNA fraction was observed in lung cancer patients compared to normal individuals (Fig. 2a–c). The Wilcoxon rank-sum test *P* values for the two groups are as follows: *P*(*CancerDetector*) < 2.2e-16, *P*(*cfSort*) = 5.919e-13, and *P*(*cfDeconvolve*) = 2.173e-06. Additionally, we plotted the receiver operating characteristic (ROC) curve and calculated the area under the curve (AUC) for disease detection (Supplementary Fig. S1), using either tumor burden or the cfDNA fraction derived from diseased tissue as the sole predictor. For lung cancer detection, the performances are as follows: AUC(*CancerDetector)* = 0.907, AUC(*cfSort*) = 0.796, and AUC(*cfDeconvolve*) = 0.695. The results indicate that *CancerDetector* surpasses all other methods in lung cancer diagnosis.

### 3.2 Liver cancer detection

We demonstrated another cancer type, liver cancer, as the second example. A significant increase in tumor burden and liver tissue-derived cfDNA fraction was observed in liver cancer patients compared to normal individuals (Fig. 2d–f). The Wilcoxon rank-sum test *P* values are as follows: *P*(*CancerDetector*) = 2.174e-11, *P*(*cfSort*) = 5.578e-10, and *P*(*cfDeconvolve*) = 9.244e-06. And the AUC values for liver cancer detection are as follows: AUC(*CancerDetector*) = 0.920, AUC(*cfSort*) = 0.890, and AUC(*cfDeconvolve*) = 0.779. *CancerDetector* again outperforms all other methods in liver cancer diagnosis, suggesting its potential to accurately diagnose various types of cancers.

### 3.3 Cirrhosis detection

In addition to cancer detection, our functions can also be applied to other disease types. Cirrhosis is a chronic liver disease characterized by fibrosis and impaired liver function, with alterations in DNA methylation patterns reflective of liver injury (Zhang *et al.* 2023). Here, we demonstrate cirrhosis detection using cfTools. We observed that liver-derived cfDNA fractions are significantly higher in patients with cirrhosis compared to normal individuals (Fig. 2g and h). The Wilcoxon rank-sum test *P* values are as follows: *P*(*cfSort*) = 6.297e-04, *P*(*cfDeconvolve*) = 6.140e-04. And the AUC values for cirrhosis detection are: AUC(*cfSort*) = 0.7381, AUC(*cfDeconvolve*) = 0.7386. Both tissue deconvolution methods exhibit reliable performance in cirrhosis detection.

### 3.4 Use case discussion

From the above three use cases, we conclude that all three methods effectively detect elevated cfDNA from diseased tissues. *CancerDetector* demonstrates superior performance in cancer diagnosis, and *cfSort* shows comparable or higher performance than *cfDeconvolve* in disease detection. Our findings show that tissue deconvolution methods (e.g. *cfSort* and *cfDeconvolve*) effectively identify elevated cfDNA fraction from diseased tissues, underscoring their clinical applications in general disease detection and monitoring. However, for diagnosing specific diseases such as cancer, incorporating known disease-specific and tissue-specific methylation patterns (e.g. in *CancerDetector*) is essential for achieving the most accurate detection outcomes. It should be noted that all three methods can be applied to detect non-cancer diseases, provided that appropriate markers are available. Additionally, *cfDeconvolve* and *CancerDetector* can be adapted to nonhuman data (e.g. mouse samples), as they do not rely on human-derived reference markers like *cfSort*.

## 4 Conclusion

We developed the R/Bioconductor package cfTools to deconvolve cfDNA from diseased or normal tissues using DNA methylation profiles. cfTools offers a streamlined workflow for sensitive cancer detection and tissue deconvolution, which can be applied in a broad range of clinical applications involving cfDNA-based diagnosis, prognosis, and disease monitoring for cancer and other conditions. We believe this toolkit will facilitate knowledge discovery from cfDNA methylation sequencing data and accelerate progress in cfDNA-based disease studies.

## Author contributions

Ran Hu (Writing – original draft [lead], Formal analysis [equal], Software [lead], Writing – review and editing [equal]), Shuo Li (Methodology [equal], Formal analysis [equal], Writing – review and editing [equal]), Mary L. Stackpole (Methodology [equal]), Qingjiao Li (Methodology [equal]), Xianghong Jasmine Zhou (Methodology [equal], Writing – review and editing [equal], Conceptualization [equal]), Wenyuan Li (Methodology [equal], Formal analysis [equal], Writing – review and editing [equal], Conceptualization [equal])

## Supplementary Material

vbaf108_Supplementary_Data

## Data Availability

The raw sequencing data of the solid tissue samples and plasma samples are publicly available under controlled access at the European Genome-phenome Archive (EGA) with the accession number EGAS00001006020 (Stackpole *et al.* 2022) and EGAD00001010880 (Li *et al.* 2023).
