# Supplementary material for: cfTools: an R/Bioconductor package for deconvolving cell-free DNA via methylation analysis
Source: Bioinform Adv. 2025 May 6;5(1):vbaf108. doi: 10.1093/bioadv/vbaf108 (PMC12124914; doi:10.1093/bioadv/vbaf108)
Supplement: vbaf108_Supplementary_Data [file vbaf108_supplementary_data.docx]

**Supplementary Data**


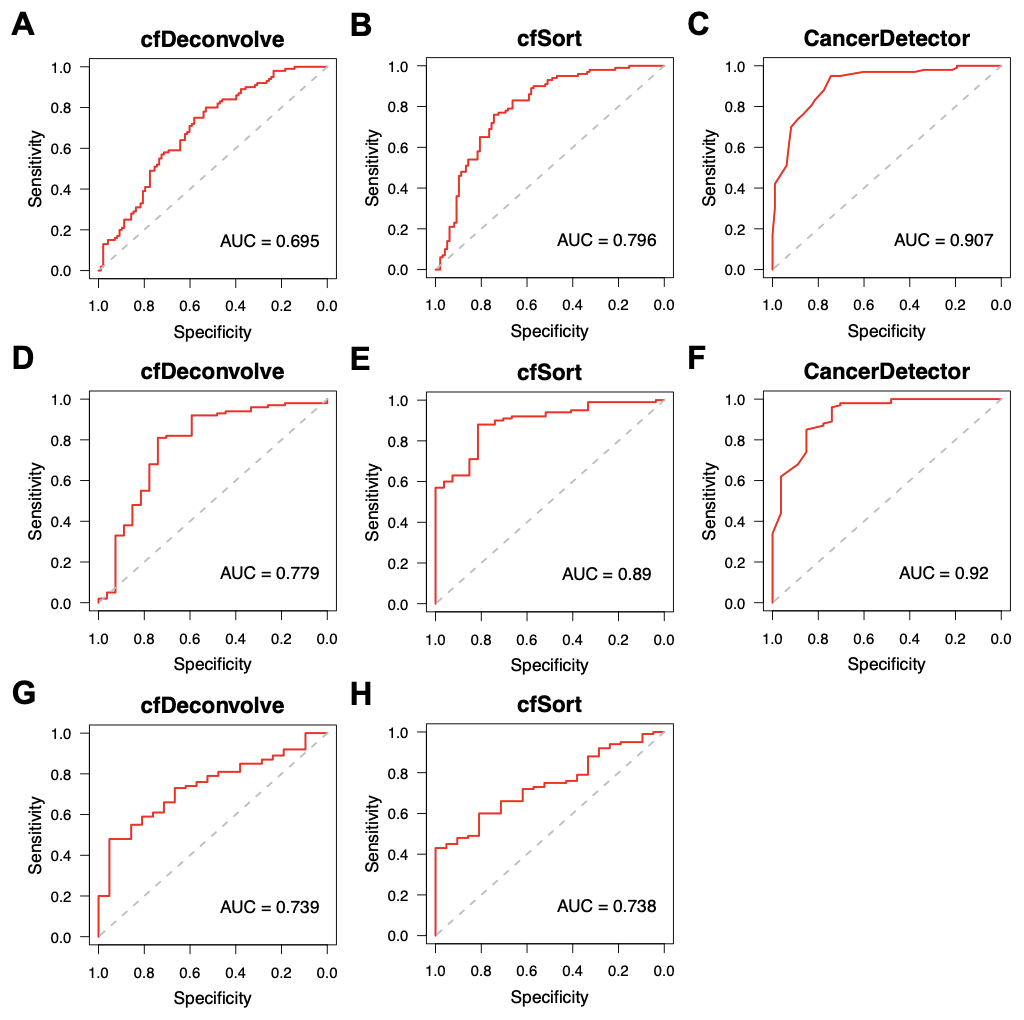


**Supplementary Figure S1.** ROC curves for disease detection using diseased tissue-derived cfDNA fractions from *cfDeconvolve* and *cfSort*, and tumor burden from *CancerDetector*. **(A-C)** Distinguishing lung cancer patients from normal individuals using estimated lung-derived cfDNA fractions and tumor burden. **(D-F)** Distinguishing liver cancer patients from normal individuals using estimated liver-derived cfDNA fractions and tumor burden. **(G-H)** Distinguishing patients with cirrhosis from normal individuals using estimated liver-derived cfDNA fractions.

**Supplementary Text S1**

Here, we provided a detailed description and the underlying algorithms of *CancerDetector*, *cfDeconvolve*, and *cfSort*.

**1.** ***CancerDetector*: Deconvolution of disease signals at the cfDNA-fragment level**

Function *CancerDetector* requires two main inputs: (1) the fragment-level methylation states of reads (column methState of the output file of *GenerateFragMeth*) that mapped to the cancer-specific markers; (2) paired shape parameters of beta distributions for cancer-specific markers. Additionally, users can tune a parameter lambda to adjust the estimated tumor burden by filtering out confounding markers that contribute disproportionately high tumor fractions. A larger lambda results in fewer markers being removed. Note that although the method was initially developed for cancer detection, it can also be applied to other diseases by replacing cancer-specific markers with disease-specific ones.

The underlying algorithms used to estimate tumor burden are described below. First, *CancerDetector* computes tumor- and normal-specific likelihoods for each cfDNA fragment by using a probabilistic model to jointly analyze the methylation states of multiple neighboring CpG sites on a single cfDNA fragment (Li *et al.*, 2018). We model the methylation pattern of a marker $k$ as a Beta distribution, i.e., $m_{k}^{T}=\mathrm{Beta}\left( {}^{T}, \rho^{T} \right)$ for the tumor class and $m_{k}^{N}=\mathrm{Beta}\left( {}^{N}, \rho^{N} \right)$ for the normal class. Given a fragment $\boldsymbol{r}=\left( r_{1}, r_{2}, \ldots r_{J} \right)$ with *J* CpG sites that falls into a marker *k*, we model the binary methylation status $r_{j}$ ($1\leq j\leq J$, 0 as unmethylated and 1 as methylated) of each CpG site $j$ to follow a Bernoulli distribution $r_{j}\sim\mathrm{Bernoulli}\left( p \right)$, where $p$ follows the methylation pattern of $m_{k}^{T}$ or $m_{k}^{N}$. Hence the class-specific likelihood of the fragment ***r*** can be calculated as

$P\left( \boldsymbol{r} | m_{k}^{T} \right)=\prod_{j} P\left( r_{j} | \mathrm{Beta}\left( {}^{T}, \rho^{T} \right) \right)$ for the tumor class and $P\left( \boldsymbol{r} | m_{k}^{N} \right)=\prod_{j} P\left( r_{j} | \mathrm{Beta}\left( {}^{N}, \rho^{N} \right) \right)$ for the normal class. With high-quality cancer-specific methylation marker set $\Omega=\{\left( m_{1}^{T}, m_{1}^{N} \right), \ldots, \left( m_{k}^{T}, m_{k}^{N} \right)\}$, we calculate the likelihoods $P(\boldsymbol{r}|m_{k}^{T})$ and $P(\boldsymbol{r}|m_{k}^{N})$ that a cfDNA fragment originates from tumor cells or normal cells.

Next, we estimate the tumor burden $\theta$(i.e. tumor-derived cfDNA fraction) for a cfDNA sample that consists of $Q$ fragments, $S=\{\boldsymbol{r}^{1},\ldots,\boldsymbol{r}^{Q}\}$, by maximizing the likelihood function $L\left( \theta\right)=P\left( S | \theta, \Omega\right)=\prod_{i=1}^{Q} P(\boldsymbol{r}^{i}|\theta,\Omega)$, $0\leq\theta<1$, where

$P\left( \boldsymbol{r}^{i} | \theta, \Omega\right)=\theta P\left( \boldsymbol{r}^{i} | m_{k}^{T} \right)+\left( 1-\theta\right)P(\boldsymbol{r}^{i}|m_{k}^{N})$.

We apply a grid search to determine the best $\theta$ that can globally optimize the likelihood function.

**2. *cfDeconvolve*: Unsupervised tissue deconvolution at the cfDNA-fragment level**

Function *cfDeconvolve* is a cfDNA tissue deconvolution approach that employs the expectation-maximization (EM) algorithm. It aggregates the tissue-of-origin likelihoods of all fragments to estimate the global composition of each tissue. The two main input files are similar to function *CancerDetector*: (1) the fragment-level methylation states of reads (column methState of the output file of *GenerateFragMeth*) that mapped to the tissue-specific markers; (2) paired shape parameters of beta distributions for tissue-specific markers. To account for the discrepancies between real-world scenarios and the predefined tissue categories, we provide a parameter emAlgorithmType to allow users include an unknown tissue type in addition to the tissue types in the reference panel. We also provide a parameter, likelihoodRatioThreshold, which serves as a cutoff to remove reads with ambiguous predictions. Reads for which the ratio of the maximum to minimum likelihoods across all tissues falls below this threshold will be excluded.

*cfDeconvolve* can deconvolve *N* tissue classes (*N* > 2) in a probabilistic manner similarly to *CancerDetector*. The goal is to infer the proportions of cfDNA that originate from *N* types of tissues, $\Theta=(\theta_{1},\theta_{2},\ldots,\theta_{N})$. Following the same principle as *CancerDetector*, *cfDeconvolve* calculates *N* tissue-specific likelihoods that a cfDNA fragment originates from each of the tissues, using the fragment-level joint methylation states (Del Vecchio *et al.*, 2021). If the fold change between the highest and the second highest likelihoods is less than a threshold (e.g., 2), the fragment is considered ambiguous and may belong to an unknown class type; otherwise, the tissue type of the fragment is unambiguous. Given a sample $S$ with all unambiguous fragments and the tissue-specific methylation marker set $\Omega$ for *N* tissue types, the maximum likelihood estimation problem is formulated as

$\max_{\Theta} P(S|\Theta,\Omega), s.t. \sum_{n=1}^{N} \theta_{n}=1,\theta_{n}\geq0$.

Assuming the independence of each fragment, the likelihood function is decomposed as the product of the likelihoods of all fragments:

$P\left( S | \Theta,\Omega\right)= \prod_{i=1}^{Q} P\left( \boldsymbol{r}^{i} | \Theta,\Omega\right)$.

For each fragment $\boldsymbol{r}^{i}$, we introduce a latent discrete variable $z_{i}$ to represent the tissue type from which the fragment is derived. The likelihood of each cfDNA fragment is formulated as

$P\left( \boldsymbol{r}^{i} | \Theta,\Omega\right)=\sum_{n=1}^{N} P(\boldsymbol{r}^{i}|\Theta,\Omega)=\sum_{n=1}^{N} P\left( z_{i}=n | \Theta\right)P\left( \boldsymbol{r}^{i} | z_{i}=n, \Omega\right)$.

Here, $P\left( z_{i}=n | \Theta\right)=\theta_{n}$ and $P\left( \boldsymbol{r}^{i} | z_{i}=n, \Omega\right)$ is the tissue-of-origin likelihood of fragment $\boldsymbol{r}^{i}$ for tissue type $n$. We denote the posterior probability of $z_{i}=n$ as $q\left( z_{i}=n \right)=P(z_{i}=n|r^{i}, \Theta, \Omega)$. According to the EM algorithm, the E-step is:

$q\left( z_{i}=n \right)=\frac{\theta_{n}P\left( \boldsymbol{r}^{i} | z_{i}=n, \Omega\right)}{\sum_{n=1}^{N} \theta_{n}P\left( \boldsymbol{r}^{i} | z_{i}=n, \Omega\right)}$, $n=1,\ldots, N$.

And the M-step is:

$\theta_{n}=\frac{\sum_{i=1}^{Q} q(z_{i}=n)}{Q}$, $n=1,\ldots, N$.

By iteratively performing the E-step and M-step with different randomly initialized values of $\theta_{n}$, we choose the final solution that gives the maximum likelihood. If users want to account for an unknown tissue type with composition $\theta_{u}=\frac{number of ambiguous fragments}{number of total fragments}$, the proportions of the known tissue types will be adjusted to $\left( 1-\theta_{u} \right)\theta_{n}$.

**3. *cfSort*: Supervised tissue deconvolution at the cfDNA-fragment level**

Function *cfSort* is the first supervised tissue deconvolution method based on deep neural network (DNN). The only input file required is the fragment-level methylation states of reads (column methState of the output file of *GenerateFragMeth*) that mapped to the tissue-specific markers. The reference marker file (cfsort_markers.txt.gz) is preloaded within the cfTools package, so users do not need to provide a marker file manually.

Given the fragment-level methylation states of a cfDNA sample, *cfSort* generates the predictions of the sample’s tissue composition across 29 predefined tissue types: $({\theta_{1},\theta_{2},\ldots, \theta}_{29})$, where $\theta_{i}\geq0$ and $\sum_{i=1}^{29} \theta_{i}=1$. The function utilizes a dual DNN ensemble implemented with TensorFlow, which is stored and loaded from the cfToolsData package. These DNN models were trained on a large and diverse dataset comprising 295,484 in-silico cfDNA samples, each generated by mixing methylation data from human tissues with a wide range of known tissue composition profiles (Li *et al.*, 2023). This supervised learning approach enables the model to robustly infer tissue proportions from cfDNA methylation patterns.

**References**

Del Vecchio,G. *et al.* (2021) Cell-free DNA Methylation and Transcriptomic Signature Prediction of Pregnancies with Adverse Outcomes. *Epigenetics*, **16**, 642–661.

Li,S. *et al.* (2023) Comprehensive tissue deconvolution of cell-free DNA by deep learning for disease diagnosis and monitoring. *Proc. Natl. Acad. Sci. U.S.A.*, **120**, e2305236120.

Li,W. *et al.* (2018) CancerDetector: ultrasensitive and non-invasive cancer detection at the resolution of individual reads using cell-free DNA methylation sequencing data. *Nucleic Acids Research*, **46**, e89–e89.
